# New avenues for residual stress analysis in ultrathin atomic layer deposited free-standing membranes through release of micro-cantilevers

**DOI:** 10.1016/j.heliyon.2024.e26420

**Published:** 2024-02-20

**Authors:** S. Burgmann, M.J. Lid, H.J.D. Johnsen, N.P. Vedvik, B. Haugen, J. Provine, A.T.J. van Helvoort, J. Torgersen

**Affiliations:** a Department of Mechanical and Industrial Engineering, NTNU, Trondheim, Norwegian University of Science and Technology, Norway; b Aligned Carbon, Santa Clara, CA, USA; c Department of Physics, NTNU, Trondheim, Norwegian University of Science and Technology, Norway; d Chair of Materials Science, Department of Materials Engineering, TUM School of Engineering and Design, Technical University of Munich, Germany

**Keywords:** Ultrathin membranes, FIB, Residual stress, Strain analysis, ALD

## Abstract

The fabrication of thinnest, yet undeformed membrane structures with nanometer resolution is a prerequisite for a variety of Microelectromechanical systems (MEMS). However, functionally relevant thin films are susceptible to growth-generated stress. To tune the performance and reach large aspect ratios, knowledge of the intrinsic material properties is indispensable. Here, we present a new method for stress evaluation through releasing defined micro-cantilever segments by focused ion beam (FIB) milling from a predefined free-standing membrane structure. Thereby, the cantilever segment is allowed to equilibrate to a stress-released state through measurable strain in the form of a resulting radius of curvature. This radius can be back-calculated to the residual stress state. The method was tested on a 20 nm and 50 nm thick tunnel-like ALD  membrane structure, revealing a significant amount of residual stress with 866 MPa and 6104 MPa, respectively. Complementary finite element analysis to estimate the stress distribution in the structure showed a 97% and 90% agreement in out-of-plane deflection for the 20 nm and 50 nm membranes, respectively. This work reveals the possibilities of releasing entire membrane segments from thin film membranes with a significant amount of residual stress and to use the resulting bending behavior for evaluating stress and strain by measuring their deformation.

## Highlights

• A FIB milling procedure allowing the release of defined micro-cantilevers from free-standing membrane-based microstructures.
• Through the release of the micro-cantilever, an ultrathin ALD  membrane's residual stress releases to measurable strain.
• Minimally invasive milling parameters allowing the release of micro-cantilevers without perturbing the initial stress state.
• Evaluated residual stress of 866 MPa and 6104 MPa for a 20 nm and a 50 nm thick  membrane structure, respectively.
• Composite FEM model maps membrane stress distribution, aligning well with out-of-plane deflection.

Through the release of the micro-cantilever, an ultrathin ALD  membrane's residual stress releases to measurable strain.

Evaluated residual stress of 866 MPa and 6104 MPa for a 20 nm and a 50 nm thick  membrane structure, respectively.

## Graphical abstract

**Figure:**
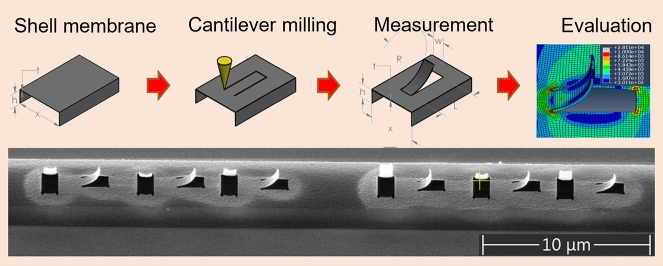

## 1 Introduction

Thin film membranes are crucial for a variety of microelectromechanical system (MEMS) devices such as pressure sensors, micro-mirrors, or nano-scale bolometers. To boost signal-to-noise, researchers are seeking free-standing membrane structures with increased aspect ratios. Thinness and dimensional stability are the key parameters to tune. However, limitations often arise due to residual stresses within thin films [1]. The magnitude of residual stress may only become apparent after releasing the structure from its underlying sacrificial support, at which point the structure may fail entirely through severe elastic or even plastic deformation. For example, the presence of post-release intrinsic stress in the structural layer of a MEMS bilayer platform causes severe deformation making the platform unsuitable for optical or frequency tuning applications [2]. Stiffening elements such as stiffening microstructures at the rim of ultrathin silicon nitride () micro mirrors [3], U-shaped trenches as basis for uncooled bolometers [4], [5], sandwiched [6] or perforated [7] membranes may ensure the integrity of the structure. Such compensating strategies may be viable, it is yet important to know and understand the amount of residual stress to compensate for [8].

Only a limited number of techniques characterize thin film properties in an isolated state, separated from the underlying substrate. Such procedures require specific architectures with fabrication steps differing from those required to make a real device [9], [10], [11], [12], [13]. As the stress state changes with every fabrication step, the residual stress of test structures may not be representative of the final device [14], [15].

To gather this information, a few sacrificial architectures in a wafer scale process could be evaluated through destructive testing, while leaving the remainder of the samples intact for further processing. This could give useful information for subsequent fabrication steps, on the condition of the membrane, and the expected behavior of the final device [16], [17]. Here, we want to measure the residual stress of specific areas of free-standing membranes through destructive testing. A cantilever-like element of the thin film gets cut out through Focused Ion Beam (FIB) milling and is allowed to relax its residual stress in measurable strain.

A few articles report on the feasibility of such FIB based approaches. The residual stress of a 300 nm thick low-pressure silicon membrane was evaluated through cut-out slots. The set dimensions of the milling step were compared to the developed dimensions of the membrane around its cut-out section [18]. Here, thicker films were under study that may induce larger changes in the dimensions. Evaluation involving image processing through differential image correlation (DIC) of SEM images was necessary, showing that residual stress can be measured through strain in membranes. Another FIB based technique, applying FIB-DIC stress analysis, uses ring-core-milling to elaborate residual stress in the structure, but is not applicable on membrane structures [19]. The possibility of cutting out defined cantilevers and more complex nanokirigami structures using FIB milling was also shown, however, not with the intention to evaluate residual stresses [20], [21]. To the best of our knowledge, FIB milling of pre-stressed ultrathin membranes to release defined micro-cantilevers of ultrathin film-based architectures below 50 nm thickness and large aspect ratios (several 100 μm^2^ unsupported area) has not been reported.

In what follows, we explore the possibilities of stress evaluation on the example of a large area single film membrane structure by FIB milling of defined cantilevers, analytical stress evaluation, and Finite Element based numerical simulation. The test geometry employed is a high aspect ratio tunnel like structure made of released ultrathin (20 nm and 50 nm thick) ALD  membranes with 500 μm^2^ unsupported area, fabricated according to a published procedure [22]. We investigate and discuss the membranes' deformation behavior and stress peaks in the structures, and deduce the residual stress built in during film growth and release.

## 2 Experimental details

### 2.1 FIB milling of defined cantilevers

**Figure 1:**
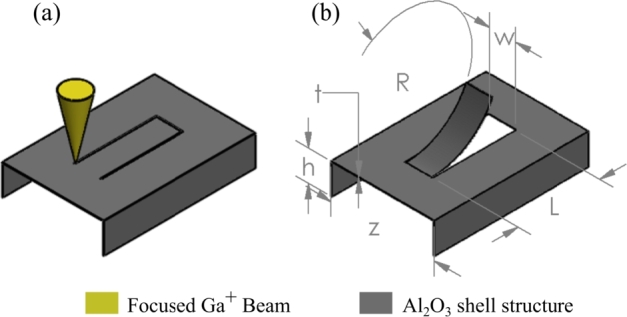
Schematic of residual stress analysis by cutting defined cantilevers from a free-standing membrane using the milling capabilities of a FIB instrument (a). After full release, the cantilever bends with a constant bending radius *R* allowing for stress evaluation of free-standing membrane structures (b). Note that the convergence angle of the cone is exaggerated for illustrative purposes.

Cantilevers with varying forms and sizes were milled from ultrathin free-standing membranes to assess the possibilities of evaluating residual stress, and specifically the proportion of intrinsic stress in free-standing thin films. The micro-cantilevers were cut from the surrounding membrane as shown in Fig. 1(a), where the ion beam is indicated with a yellow cone. The free-standing ALD  membranes employed as test objects had structure dimensions as indicated in Fig. 1(b). The height (*h*) was 600 nm, the width (*z*) was , and the thickness of the constituent membrane (*t*) was 20 nm and 50 nm, respectively. Details for fabricating the test structures are explained elsewhere [22]. Cantilevers were cut from the top membrane of the tunnel like structure as indicated in Fig. 1(b). The milling itself was performed in a dual beam FIB instrument (Helios G4 UX, Thermo Fisher Scientific, Massachusetts, US) with perpendicular milling angle of the Ga^+^ beam. As milling effects such as Ga^+^ implantation, swelling, or lattice distortion are known phenomena that can lead to unwanted strain in the exposed material [23], [24], [25], the effects of Ga^+^ implantation were considered in the choice of the milling strategy. Simulations of a 30 kV Ga^+^ beam with a silicon substrate indicate that the impact of ion implantation and lattice dislocations are confined to an area of approximately 80 nm in depth and width from the FIB focal point [26]. Based on this, we can expect that aside from areas in proximity to the milled edges, the cantilever will not be subject to ion beam introduced strain that could alter the resulting bending curvature. Hence, the measurable deformation occurring after milling is predominately caused by residual stress present in the thin film. This is a crucial difference to the FIB fabricated nanokirigami structures, where global ion beam irradiation is used to deliberately modify film properties and introduce stress through material swelling on a large sample area, and hence cause bending of the cut cantilevers [27]. The milling step was conducted by moving the Ga^+^ beam along a defined pathway while being focused on the membrane. To determine beam settings, a parameter study was undertaken and milling parameters facilitating complete release of the entire membrane segment with only minimal in-plane deformation were chosen. While beam parameters of 30 kV and 2.6 pA were sufficient to cut through the thin  membranes, these settings occasionally resulted in only a partial release, rendering uncontrolled deformation of the membrane segment. Further optimization was done in the milling settings, where the dwell time was extended from  to  using 50% point overlap. Calibrated to the standard (111) silicon material profile of the instrument, the milling depth was set to 200 nm, which was sufficient to cut through the membrane completely. To avoid unexpected distortion of the segment prior to completely cutting the cantilever, the three individual line segments were milled in parallel mode. Here, all lines are milled in an iterative sequence, where each line is thinned to the same depth before returning the ion beam to the first line. The process is repeating until the anticipated milling depth is reached, and the cantilever can deflect freely.

### 2.2 Measurements and characterization

Imaging and measurement of the bending curvature were done by scanning electron microscopy (SEM) using the electron beam imaging capabilities of the dual beam FIB. Beam settings for imaging with 0.2 nA current and 5 kV acceleration voltage were used together with a through lens detector (TLD). Measurements of the bending curvature, as well as the projection length (*x*) and out-of-plane deflection of the bent cantilever beams, were done using the taken SEM images, where the perspective view and tilt angle of 32°were considered. The observed bending curvature was used as a physical model for further evaluations. The bending curvature of the cantilevers indicates a strong through plane stress gradient in the thin film before cutting. Analytical calculations of the present stress were performed under consideration of the change in strain through the thickness of the thin film. A complementary simulation of the stress distribution in the membrane was done in Abaqus for finite element analysis (FEA) [28], where the observed bending curvature was used to verify the consistency of the model. The evaluation of residual stress via the eigenstrain method is an established approach [29]. For modeling in the commercial software Abacus, a combination of the thermal expansion coefficient and a temperature step was used to mimic the inelastic strains and to derive the residual stress in the simulated cantilever. A composite model was used to simulate the deformation behavior of the released cantilevers, where the thermal expansion coefficient was employed as a pseudo-thermal to mimic the presence of inelastic strains that are the actual cause of residual stress. Inelastic processes resulting from the growth mechanisms or a mismatch of thermal expansion between the layer and the substrate led to eigenstrain indicating a permanent strain in the thin film. The resulting bending curvature in the simulation was compared to the observed bending curvature in the experiment to assess the correspondence of the modeling results. Further details on the used model and material parameters are given in chapter 3.4, where the simulation results are presented.

## 3 Results and discussion

FIB based release of micro-cantilevers from free-standing single film membrane structures can have applicability in fabrication and development processes, where understanding residual stress is important to manufacture reliable devices. When investigating the resulting structures we need to understand:

1. If FIB milling can be applied on pre-stressed thin films to release defined micro-cantilevers of various sizes.
2. The optimal dimensions of FIB milled micro-cantilevers for residual stress evaluation.
3. The dependency of cantilever length on the reliable measurable deformation.
4. If the response of the cantilever can be used as a physical model for residual stress evaluation of a free-standing membrane consisting of a few nm thick thin film.

To address these issues, a set of rectangular-shaped micro-cantilevers were cut from ultrathin membrane structures made from ALD  thin films, and the resulting bending behavior was studied. In an initial experiment, test structures with 20 nm and 50 nm membrane thickness were examined regarding the impact of different stress cases on the applicability of milling micro-cantilevers from pre-stressed membranes. We are interested in the acceptable range of milling parameters, bending curvature, and reproducibility of the method. After establishing milling parameters that support successful micro-cantilever release, the corresponding residual stress was evaluated for each thickness based on the observed bending curvature. Complementary FEM simulations were conducted, allowing an estimation of the residual stress present in the free-standing thin film.

### 3.1 FIB milling of pre-stressed micro-sized cantilevers

Thin film membranes are often brittle and subject to a significant stress gradient through the layer, making it difficult to successfully mill defined structures without causing destruction to the membrane. As residual stress is related to the growth mechanism and the process parameters, it can be considered the sum of extrinsic and intrinsic stresses. While extrinsic stress is commonly resulting from a mismatch of thermomechanical properties and thus strongly dependent on the process conditions [30]. Intrinsic stress is related to the growth mechanism and thus a thickness dependent variable. When milling pre-stressed free-standing membranes, the bending direction should be dependent on the through plane stress gradient and not affected by the milling process. To verify this, ALD  and PECVD  membranes were tested. ALD  is generally under tensile stress while attached to a substrate [31], while PECVD  is reported to be under compressive stress while attached to the substrate [32]. The additional experiment showed that in contrast to the ALD  membranes, free-standing  membranes bend downward. From this we can conclude that the bending direction is determined by the intrinsic film properties and not influenced by the FIB milling process.

**Figure 2:**
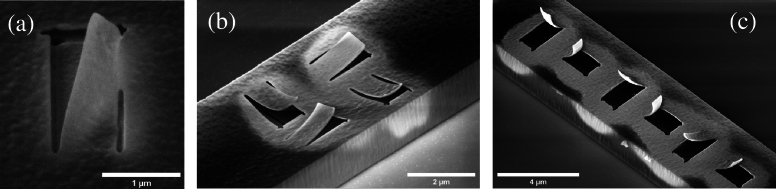
(a) Partially milled micro-cantilever deforming before full release is reached; (b) micro-cantilevers milled from a 20 nm thick membrane; (c) micro-cantilevers milled from 50 nm thick membrane.

If isotropic residual stress relaxes in strain upon the removal of constraints, the released fragment of the thin film should theoretically expand or contract equally in all directions. To verify whether the residual stress is isotropic, and no other effects than the residual stress cause the bending of the cantilever, the bending behavior should be independent of the orientation of the cut-out section. Indeed, two cantilevers with different orientations (perpendicular and longitudinal to the length of the membrane) show a matching bending curvature (see Fig. 2 (b) and (c)). The membrane contracts equally in all directions, therefore a bending of the released cantilever perpendicular to its length is occurring that indicates the tendency of the released section to equilibrate in a spherical shape [33]. Various length to width ratios of the cantilever were examined, where the different bending curvatures led to matching stress levels. Impacts of other nonlinear modes of deformation asides from pure bending were not observed in structures below 5 μm length. Above this threshold, inconsistencies in the resulting bending curvature were observed. We expect the radius of curvature for films with the same thickness to be independent of the cantilever length. Milling free-standing membranes in a pre-stressed state can however, lead to distortion in the film before completely releasing the cantilever or even rapture of the entire membrane. The cantilever would deform during milling, resulting in a change in focus along the milling pathway. This could affect the milling rate during processing, leaving the milled pathway partially uncut. To identify ideal milling parameters, an initial milling experiment was conducted where exposure dose, dwell time, and milling depth were varied. Indeed, a partial cut of the predefined milling path occurred in some instances. Fig. 2(a) demonstrates a partially released cantilever milled with 180 nm milling depth and  dwell time. The cantilever remains locally connected to the surrounding material and thus distorted. To establish a symmetric release and thus avoid higher modes of bending or bifurcation in non-symmetric distorted, partially connected cantilevers, serial and parallel milling sequences were evaluated. Parallel milling sequence consists of several milling passes, where the membrane was thinned down until a quasi-simultaneous release of the cantilever was achieved. This approach resulted in a reliable symmetric release of the defined cantilevers. To further reduce the risk of non-symmetric release, long milling pathways and thus large cantilevers should be avoided. However, visual analysis of the cantilevers after milling shows that the area surrounding the milled cantilevers appears brighter, suggesting a FIB related alteration of the membrane. Although Ga^+^ ion implantation can cause contrast changes in milled samples [34], it is unlikely the source of the here observed ‘shadow’ surrounding the cantilevers. This assumption was derived from the milling settings, where only local milling was applied, and global irradiation was strictly avoided. Local milling is based on a point-focused ion beam on the membrane, affecting matter below the point of impact. For a silicon sample, the impact depth is within a radius of about 80 nm [34]. In case of ALD  a lower impact depth is likely as the material density is higher. This indicates that a direct effect by ion beam exposure is restricted to the exposure radius of approximately 80 nm surrounding the focal point. The observed brightness, which is much larger and also extends over the side walls of the tunnel like structure (Fig. 2(b)and (c)), must therefore be due to another phenomenon. A more likely explanation could be seen in a milling depth exceeding the thickness of the membrane, thus milling into the substrate material inside the structure. Here, atoms sputtered from the substrate material inside the tunnel are trapped and cannot escape as on a free surface. Thus, redeposition of sputtered atoms occurs from the inside of the tunnel, leaving a thin film coating on the membrane. This also explains what can be seen in Fig. 2(a), where partial milling of the underlying silicon substrate is observed in the top left corner.

### 3.2 Cantilever dimensions

**Figure 3:**
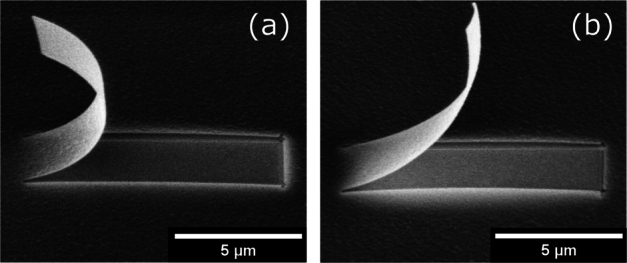
Inconsistency of bending radius for long cantilevers with 10 μm length milled from a 50 nm membrane (a) bending radius 2.2 μm (b) bending radius 5.7 μm.

**Figure 4:**
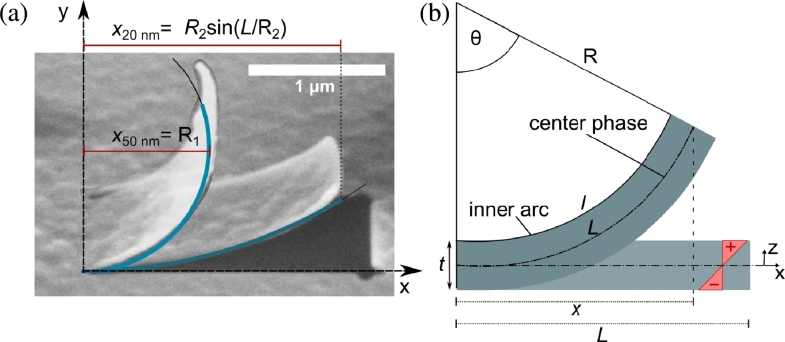
(a) Illustration of thickness-dependent residual stress in membranes with 20 nm and 50 nm thickness by overlaying cantilevers with corresponding dimensions, yet different membrane thickness. (b) Cross-sectional bending model of the cantilever before release (flat) and after release (bend).

To evaluate the limits and possibilities of FIB milled micro-cantilevers for the purpose of obtaining intrinsic properties of membranes, cantilevers were milled and released from a free-standing membrane. The results show a measurable radius of curvature that can theoretically be used as a physical model for stress analysis. A set of rectangular cantilevers with a width (w) of 0.8 μm and length (L) of 0.4 μm, 0.8 μm, 1.2 μm and 1.6 μm, 5 μm and 10 μm were manufactured to evaluate the influence of the cantilever length on the bending curvature. Cantilevers up to a length of 5 μm showed a reproducible bending curvature, where the measurable projected lengths followed the relation $x=Rsin(\frac{L}{R})$ independent of L. The respective standard deviations were 0.04 μm and 0.08 μm for the 20 nm and 50 nm thick membranes, respectively. When comparing the bending radius of short cantilevers with 0.4 μm length to the evaluated radius of longer cantilevers, the measured curvature seems to correspond. Yet, the evaluation of the bending radius based on the difference between cantilever length (*L*) and projection length (*x*) indicated in Fig. 4 (b) is difficult due to a small length difference that is at the limit of the measurable accuracy. On the other hand, long cantilevers with 0.8 μm width and 10 μm length showed a high inconsistency of the resulting bending curvature. For these cantilevers, a significant deviation was observed, as shown in Fig. 3 (a) and (b). Here, the equilibrium state may not be reached instantly, but deformation may continue over an unknown period of time. To evaluate the effect of continuous deformation over an extended period of time, further experiments are required, which are however not a subject of this study.

It seems that the bending curvature of the cantilevers coincide well regardless of their length, indicating that the impact of higher order deformation events such as buckling, or bifurcation is minor for the investigated cantilever lengths. As the measurement of short structures was difficult due to the limited deformation and the large impact of the compressive tensile strain, we chose cantilevers with a length of 1.6 μm to evaluate the residual stress in the 20 nm and 50 nm thick films.

### 3.3 Growth-generated stress in ALD deposited thin films and resulting bending curvature

Since free-standing membranes are usually either constrained by a surrounding structure or part of a larger multilayer device, the effects of residual thin film stress could stay undetected during the manufacturing process or only become visible at a later stage of the fabrication process. Our underlying hypothesis is that this built-in stress is observable via the elastic response or deformation of the sample when released from its constraints. The release of a small membrane segment from its surrounding support would immediately deform according to its built-in stress, resulting in a measurable deformation. This is a common strategy for measuring residual stresses in the macroscopic domain [35]. In our case, a significant upwards bending can be observed for all our released cantilevers (Fig. 3). Apparently, a significant through plane stress gradient is present in the membrane, where the structures' bending along a circular curvature suggests that the stress is linearly distributed over the film thickness. In an ALD process, thin films are deposited in a layer-by-layer sequence in a self-saturating manner. Only a slight change in strain for each subsequent layer can cause a significant amount of stress in the resulting film, thereby imposing a bending moment on the resulting structure when released [11]. This characteristic suggests that the resulting bending force is thickness dependent and influenced by the deposition parameters such as temperature, pressure, chemistry, and number of cycles [36]. Indeed, it is reported that the residual stress for 100 nm thick ALD  thin films can be as high as 322 MPa [37] when deposited at 200 °C. Our study supports this observation. Cantilevers milled from 50 nm thick membranes have bending curvatures with radius of 1 μm, whereas cut out cantilevers from 20 nm membranes show measurable bending curvatures with radius of curvature of around 3 μm. Confirming the thickness dependency, where cantilevers with 50 nm thickness resulted in a smaller bending radius compared to cantilevers with 20 nm thickness, indicating an increasing level of stress with membrane thickness.

Based on the bending radius as measured by SEM imaging, an evaluation of the underlying stress was conducted. In a simplified assumption, the deformation phenomena are caused by a linear change in strain through the film thickness, where strain is assumed constant in the in-plane direction. Before milling, the residual stress in the membrane changes from bottom to top with the neutral phase being the axis where the relative compressive stress from the bottom and the relative tensile stress from the top equilibrates. In the scenario where half of the layer is under tensile stress and half of the layer is under compressive stress, the neutral axis would be in the center between the top and bottom layers of the film. However, since the deposition conditions are the same during growth and the evaluated stress in the layer appears to increase faster than linear with film growth, we can assume that the center phase is not neutral. After milling, stresses are released, resulting in a strained membrane segment that follows a constant radius of curvature. Calculation of the membrane strain was done based on the bending radius of the released cantilevers. However, since the intrinsic strain in the cantilever appears to have a higher tensile share, this could result in a shortening of the entire cantilever after releasing the membrane segment. To evaluate the impact of releasing residual stress on the cantilever length (L) measurements were done before and after milling. Measurements were done on the 10 μm long cantilevers milled from the 50 nm membrane. The cantilever length was determined from the curled cantilever after milling and compared to the set cantilever length. The results show a 10% shortening of the overall length from 10 μm to 9 μm. The shortening seems to be dependent on the tensile stress present in the membrane upon release since there is no measurable change in cantilevers with 20 nm membrane thickness. We consider the overall effect to be negligible for cantilevers with a length of 1.6 μm. Evaluating the curvature is expected to give an indication of the through plane stress gradient on the membrane prior to the release. The curvature measurements for the different cantilever segments and membrane thicknesses are shown in Table 1. To illustrate the thickness dependency of the residual stress on the bending curvature, cantilevers with 20 nm and 50 nm thickness were overlaid in Fig. 4(a). From the illustration, it becomes clear that the radius of curvature for the 50 nm layer (R_1_) corresponds to the projection length ($x_{\text{50 nm}}$), while the radius of curvature for the 20 nm membrane ($R_{2}$) cannot directly be measured but has to be calculated based on other measurable variables. Calculation of the bending radius for the 20 nm membrane was done using the total length (*L*) and the projection length (*x*) shown in Fig. 4(b) [38]. If the projected length follows the relation $x=R\sin\left(\frac{L}{R}\right)$ independent of the cut-out length and width, the magnitude of the through plane stress gradient should remain constant, and one could assume that the through plane stress gradient is equibiaxial. One could arrive at the conclusion that the measure of the curvature is indicative of the through plane stress gradient if the same stress magnitude can be evaluated from structures with different lengths. Thus, in the analysis, the film is assumed to be of uniform thickness, and stress is equibiaxial and constant throughout the film thickness. The maximum stress in the membrane ($\sigma_{\text{m}}$) was derived from the bending radius, using the change in strain between the center phase and the inner arc. The length of the center phase is equal to the total length of the cantilever (*L*). Evaluation of the intrinsic strain in the membrane ($\varepsilon_{\text{m}}$) was derived from geometric relations in the bent state. As indicated in Fig. 4(b), we assume that *θ* is the angle of the arc and *L* is the length of the center phase. While the length of the center phase (*L*) stays independent of the bending radius, the length of the inner arc (*l*) shortens during bending. Thus, in the bent state, *L* is greater than *l* (L≥l). Derived from the geometric relations, the length of the center phase can be expressed as L=Rθ, while the length of the inner surface can be expressed as $l=(R-\frac{t}{2})\theta$. By using these expressions in equation (1), the strain in the membrane ($\varepsilon_{\text{m}}$) can be calculated as follows:

(1) $$\varepsilon_{\text{m}}=\frac{L-l}{L}=-\frac{t}{2R}.$$

**Table 1:** Measurements and calculated bending radius of cantilever beams with  length and  width.

Membrane thickness [nm]	20 nm	50 nm
Projection length *x* [μm]	1.50 ± 0.04	1.04 ± 0.08
Radius *R* [μm]	2.93 ± 0.77	1.04 ± 0.08

Strain *ε*	0.0034	0.024
Residual stress *σ*_m_ [MPa]	866 ± 227	6104 ± 483

After evaluating the maximum intrinsic strain ($\varepsilon_{\text{m}}$) based on the bending radius, Hook's law was applied to estimate the maximum residual stress ($\sigma_{\text{m}}$) in the membrane. Resulting from the growth process, the membrane is under equal biaxial stress, where the same misfit strain is generated simultaneously in all in-plane directions. The resulting equal biaxial stress state was regarded in the stress calculation, where the biaxial version of the Young's modulus was applied. The following equation was used in the calculation:(2)
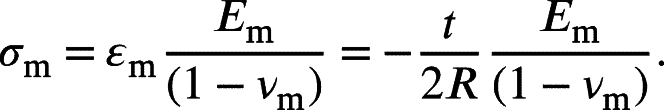


Here $\frac{E_{\text{m}}}{\left(1-\nu_{\text{m}}\right)}$ is the biaxial elastic modulus of the ALD  membrane with $\nu_{\text{m}}$ being the Poisson's ratio of ALD , *R* is the bending radius and *t* is the thickness of the membrane. Stress calculation was done based on cantilevers with a length of , corresponding to a bending radius of  ± 0.7 μm and  ± 0.08 μm for the 20 nm and 50 nm cantilevers, respectively. In total, 9 cantilevers for each membrane thickness were evaluated. The measurement results for the projection length indicate a high reproducibility with a standard deviation of 0.04 μm and 0.08 μm for the 20 nm and 50 nm membrane, respectively, the calculated radius of curvature for the 20 nm membrane shows a significantly higher standard deviation of 0.7 μm. Calculation results for residual stress in both membranes ($\sigma_{\text{m}}$) are shown in Table 1. Material parameters of ALD  used in the calculations to estimate the residual stress ($\sigma_{\text{m}}$) in the membrane are E_m_ = 195.3 GPa, $\nu_{\text{m}}$ = 0.24,  = 4.2 ppm/°C. We deducted a maximum stress of  ±  and  ±  for the 20 nm and 50 nm membrane, respectively. While the stress evaluated for both the 20 nm and 50 nm membrane coincides well with published measurements [14], [16], a comparatively high stress estimate is published for a 20 nm thick membrane with  ±  [16]). Reasons for this discrepancy could be seen in the difficulties of evaluating the stress in such ultrathin films while they are still attached to an underlying substrate. In this case, the mismatch of thermomechanical properties between the different layers could impact the final results. Further studies are necessary to understand the reasons for the divergent result found in literature.

It has to be noted that the observed deformation after release occurs without any applied external forces to the cantilever, and thus supports our hypothesis of pure bending due to a through plane stress gradient introduced into the material during film growth. To evaluate the origin of this built-in stress, we discuss the following two mechanisms: (I) extrinsic stress resulting from a mismatch of thermomechanical properties between substrate and thin film and (II) growth-generated intrinsic stress [39]. While extrinsic stress can be calculated based on the thermal expansion coefficient of the materials present, intrinsic stress must be deducted through measurable properties such as deformation and load via a suitable mechanical model [40]. In most cases, intrinsic stress is the domination portion of the max residual stress in the thin film structure. To calculate the amount of intrinsic stress, the extrinsic (thermal) stress has to be subtracted from the evaluated residual stress in the structure. When evaluating the presence of stress due to thermomechanical properties, the constraints are removed during etching of the underlying sacrificial layer, which then can no longer impact the film properties. However, an evaluation of the thermally introduced stress at the interface can give insight into the extent of the stress components in the thin film prior to release. For an evaluation of the mismatch in thermal expansion, the deposition, and growth parameters of the relevant layers are considered. In the manufacturing process of the presented test structures, an ALD  etch stop layer was deposited on a sacrificial  layer, and in a later step, the sacrificial  was removed in a vapor HF etch step. For an evaluation of the introduced stress at the interface, both the  and  layer are considered [22]. The thermally generated stress ($\sigma_{\text{th}}$) is then calculated as follows:

(3) $$\sigma_{\text{th}}=\Delta \alpha_{\text{th}}\Delta T\frac{E_{\text{m}}}{\left(1-\nu_{\text{m}}\right)}.$$

where $\Delta \alpha_{\text{th}}$ is the difference in the thermal expansion coefficient of the underlying sacrificial  layer and the  thin film, $\Delta T_{\text{th}}$ being the temperature step between deposition temperature and room temperature (225 °C), $E_{\text{m}}$ is the elastic modulus of the ALD  film, and *ν* the Poisson's ratio of the  film. The parameter for the thermal expansion coefficient of  deposited by PECVD used in this calculation was  = 0.4 ppm/°C. The difference in thermal expansion coefficient was calculated as  and used to calculate the thermally generated stress in the film (Eq. (3)). The calculation gives a thermally generated stress of . Based on this calculation, we can conclude that thermally introduced stress, and hence the mismatch between material properties seems to be of minor share importance for the maximum stress ($\sigma_{\text{m}}$) calculated from the bending curvature. However, the thermomechanical properties appear to have a greater impact on thinner films, accounting for 12.9% compared to 1.8% of the total stress for the 20 nm and 50 nm membranes, respectively. Based on this analytical evaluation, we can argue that both, the 20 nm and 50 nm membranes, have a notable amount of intrinsic stress as a result of the growth mechanisms during ALD. While the analytical calculation of the intrinsic properties in the thin film gives an estimate of the maximum residual stress present, it does not give information on the stress distribution in the free-standing structure. However, unwanted residual stress will ultimately lower the resilience of the structure and show some degree of deformation, that can become visible during the fabrication process. Thus, the development of reliable fabrication processes necessitates knowledge of the impact of residual stress and its impact on the flatness and integrity of the resulting membrane structure. To assess this, stress simulation derived from the bending curvature of FIB milled micro-cantilevers was conducted.

### 3.4 Intrinsic strain modeling and stress analysis

**Figure 5:**
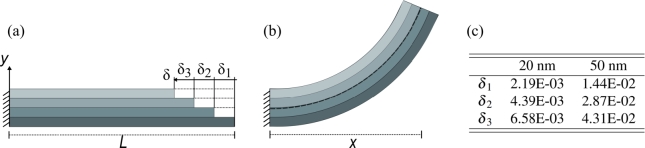
Schematics and table showing: (a) Layer model with detached layers resulting in in-plane contraction of each layer; (b) Layer model with connected layers resulting in bending curvature along a curvature; (c) Table with strain values assigned for each layer in Abaqus for both evaluated film thicknesses.

Generally, residual stress has an influence on the integrity and stability of free-standing structures. Especially, for structures with large overhangs, residual stress can have a detrimental effect on the entire structure and thus on the final device. However, the origin of thickness-dependent residual stress is less obvious without a physical model explaining the underlying phenomena during film growth [41]. To reduce the effect of stress, knowledge of both the maximum stress and the stress distribution in the structures is essential and can be obtained via FEM which captures the radius of curvature of the released cantilever. The modeling approach was derived from the sequenced ALD process, where a sublayer is deposited within each cycle, increasing the film thickness. Considering the self-limiting nature of the ALD process, we can reasonably assume that each sublayer has the same thickness. Our observation of the bending behavior in the micro-sized cantilevers indicates that the driving forces for deformation are a linearly distributed strain over the thickness of the material, together with a constant strain in the in-plane direction. We can argue that each sublayer deposited during one cycle of an ALD process is subject to a change in strain, and thus could be seen as a layer in a composite model with a specific strain in the in-plane direction. We hence assume that the membranes consist of a number of layers with the same material properties (e.g. elastic modulus and Poisson's ratio), but varying strain in the in-plane direction. Hence, if isolated from the adjacent layers, each layer contracts only in the in-plane direction indicated by a contraction *δ* shown in Fig. 5(a).

**Figure 6:**
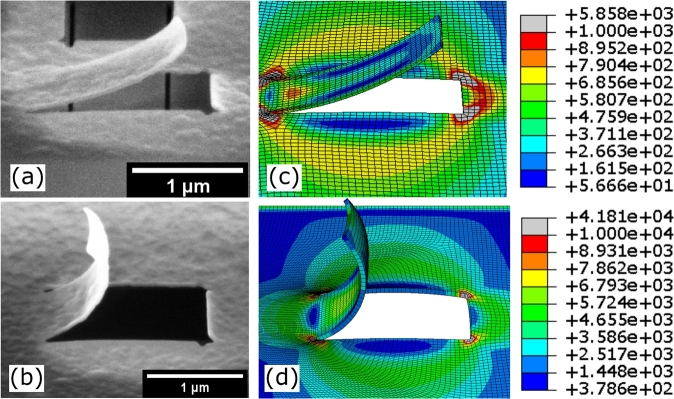
Comparison of SEM images and FEM model showing the cantilever deformation and stress distribution in the resulting structure, where the maximum stress is found surrounding the edges of the surrounding membrane. (a) 20 nm membrane after FIB release, (b) 50 nm membrane after FIB release; (c) FEM model of 20 nm membrane (units in MPa); (d) FEM model of 50 nm film (unit in MPa).

In the case of a permanent bond between the layers, the compressive and tensile forces within the layers will be transformed upon release into micro-cantilever bending along a circular curvature with constant radius *R* and projection length *x* (Fig. 5(b)). In an equivalent layered numerical model, the number of layers can be seen as the mesh in the *y* direction. To evaluate the stress distribution in the membrane, we chose a fitting model approach, where the strain calculated from the experimentally observed bending curvature in the SEM images is used to determine the input variable assigned to each layer. The numerical projection length *x* and the bending curvature of the modeled cantilever are compared to the experimentally observed values for verification. For a cantilever with a length of , the strain between the center phase and the inner arc of the bent membrane was calculated as $\varepsilon_{\text{20 nm}}=3.4\cdot 10^{-3}$ and $\varepsilon_{\text{50 nm}}=2.4\cdot 10^{-2}$ (Eq. (1)). Corresponding to the calculated strain, a FEM model was created, using the composite modeling approach. Each layer within the composite model was assigned a specific strain. Initial tests with 2 to 20 layers showed that increasing the number of layers above 4 layers does not significantly improve the modeling results. However, more layers add to computing resources and time. This is related to strain assignment in Abaqus [27]. Although strain is a well-known physical phenomenon, assigning a specific strain value to a certain layer is only possible by applying subroutines in Abaqus. However, the uniform contraction or shrinkage of matter due to growth-generated stress does not differ in its physical effect from thermally introduced strain. For this reason, a more readily available solution for modeling is to use the coefficient of thermal expansion together with a temperature change as a tool to assign a specific strain to each layer within the material properties of the layer. Derived from the initial test, a FEM model with 4 layers was investigated, where the strain was assigned in the material properties corresponding to the calculated strain. Strain values used in this model are shown in Fig. 5(c) and correspond to the change in strain through the thickness of the membranes. The results of the modeled cantilever structures are shown in Fig. 6, where cantilevers with  length and thickness of 20 nm and 50 nm are displayed, and strain results are compared to the results acquired from the SEM images taken after FIB milling. Fig. 6(c) and (d) present the Von Mises stress contour of the modeled cantilevers with film thickness of 20 nm and 50 nm, respectively. The comparison of observed and simulated cantilevers shows a good correlation of the out-of-plane deformation, with 97% and 90% agreement for the 20 nm and 50 nm membranes, respectively. It has to be noted, that while for the 20 nm membrane, the deflection and projection length *x* coincides well with the FEM model, the strong warping of the edges of the membrane segment in the 50 nm cantilevers is not entirely reproduced in the modeled cantilever. However, with only a slight deviation of projection length *x* and total deformation, it can be concluded that using a composite model together with a specified strain on each layer, a reproduction of the overall deformation behavior in the membrane is feasible. The results provide a reasonably accurate picture of the stress distribution prior to release. In Fig. 6, the stress distribution in the milled micro-cantilevers is indicated by a color map with unites given in MPa, where stress peaks are located at the corners of the surrounding membrane. Low-stressed areas can be seen where stress was released through strain, as expected. Such a procedure produces results that can potentially be used during device development to constitute engineered solutions and reduce fabrication time, waste, and repetition of processing steps due to mechanical failure.

To summarize, we demonstrate the successful FIB milling of micro-cantilevers for the analysis of residual stress in pre-stressed thin film ALD  membranes with thicknesses of 20 nm and 50 nm. Micro-cantilevers with length between  and width between  were milled, resulting in an immediate stress release, where the cantilevers bent along a circular curvature with constant radius to reach a relaxed state. Based on the radius of curvature, both analytical and simulated analysis of the residual stress prior to release are shown, allowing for rapid analysis of samples with various sizes and membrane thicknesses. The evaluated residual stress is in line with values presented in the literature. However, there are limitations to the length of the micro-cantilevers in the context of the reproducible measurement of the resulting bending radius. Based on our evaluation of various cantilever dimensions, cantilevers with length of 1.6 μm and width of 0.8 μm were selected for these specific film thicknesses and deposition conditions. Due to the freedom in design by both placement and dimensions of FIB milled cantilevers with the presented approach, residual stress can be precisely analyzed at any point during the manufacturing process. A thickness dependency, where stress increases with thickness, is reflected in the six times higher through plane stress gradient for the 50 nm layer compared to the 20 nm layer. A good correlation between the observed and simulated results is achieved by using the analytically calculated strain as the input variable for the FE simulation and comparison of the resulting stress state in the architecture.

## 4 Conclusion

We investigated the possibility to evaluate residual stress of free-standing tunnel like  membrane structures through the controlled release of a micrometer scaled cantilever beam by means of a focused ion beam milling (FIB) step. The method was performed on structures, with a membrane thickness of 20 nm and 50 nm, respectively. As such membranes are often brittle and subject to a significant stress gradient through the layer, the milling parameters are decisive for successful release. Milling parameters are presented that facilitate the relaxation of a cut-out cantilever, while avoiding destruction and distortion in the cantilever segment. Strain analysis utilizing the acquired milling parameters and the observed bending curvature caused by an instant stress release through strain is shown. Various cantilever dimensions were evaluated to circumvent the impact of other deformation modes than bending. Based on the bending curvature of cantilevers with length of 1.6 μm, a residual stress of 866 MPa and 6104 MPa was estimated for the 20 nm and 50 nm thick membrane, respectively. The values obtained coincide well with experimental values found in the literature. The thermally introduced extrinsic stress can be estimated to 112 MPa suggesting that the amount of intrinsic stress generated during film growth plays the dominant role in the total residual stress. In a finite element (FEM) analysis, a composite model with 4 layers was built to simulate a linearly distributed stress over the film thickness, as well as a constant strain in the in-plane direction, which corresponded well with the observed deformation behavior of the thin film cantilever upon release. This model allowed to assess critical regions in the thin film architecture. The presented approach can be employed on sacrificial architectures on a wafer and has likely applicability in small-scale development processes to monitor the intrinsic properties during and post fabrication to ease monitoring and quality assurance.

## CRediT authorship contribution statement

**Stephanie Burgmann**: Conceptualization, Data curation, Formal analysis, Writing – original draft. **Markus Lid**: FIB milling, Writing – review & editing. **Håkon J. D. Johnsen**: Formal analysis, Writing – review & editing. **Nils Petter Vedvik**: Methodology, Software. **Bjørn Haugen**: Supervision, Writing – review & editing. **J. Provine**: Methodology. **Antonius T. J. van Helvoort**: Supervision, Methodology, Writing – review & editing. **Jan Torgersen**: Conceptualizion, Supervision, Funding, Writing – review & editing.

## Declaration of Competing Interest

The authors declare the following financial interests/personal relationships which may be considered as potential competing interests:

Jan Torgersen reports financial support was provided by The Research Council of Norway.
